# Rapid Measurement of Lactate in the Exhaled Breath Condensate: Biosensor Optimization and In-Human Proof of Concept

**DOI:** 10.1021/acssensors.2c01739

**Published:** 2022-11-21

**Authors:** Shulin Zhang, Yu-Chih Chen, Alaa Riezk, Damien Ming, Lidiia Tsvik, Leander Sützl, Alison Holmes, Danny O’Hare

**Affiliations:** † Department of Bioengineering, Imperial College London, LondonSW7 2AZ, U.K.; ‡ Faculty of Medicine, Department of Infectious Disease, Centre for Antimicrobial Optimisation, Imperial College London, LondonSW7 2AZ, U.K.; § Laboratory of Food Biotechnology, Department of Food Science and Technology, BOKU-University of Natural Resources and Life Sciences Vienna, Muthgasse 11, WienA-1190, Austria

**Keywords:** lactate biosensor, exhaled breath condensate, non-invasive detection, Prussian blue, lactate oxidase, clinical diagnostics

## Abstract

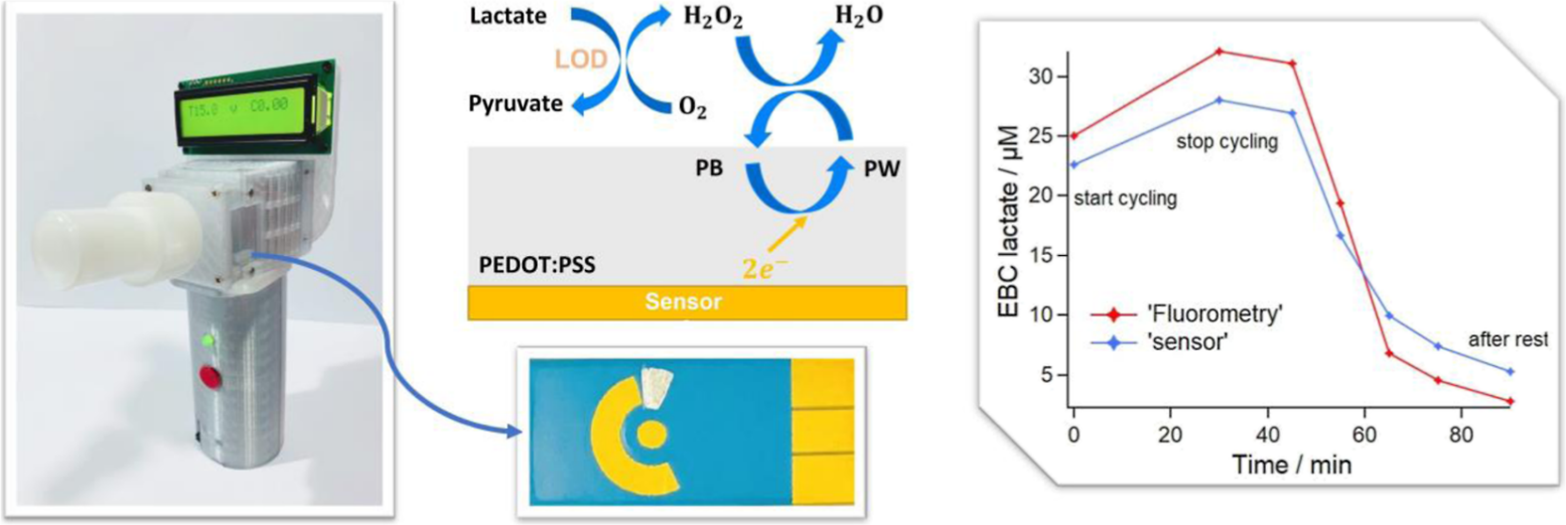

Lactate concentration is of increasing interest as a diagnostic for sepsis, septic shock, and trauma. Compared with the traditional blood sample media, the exhaled breath condensate (EBC) has the advantages of non-invasiveness and higher user acceptance. An amperometric biosensor was developed and its application in EBC lactate detection was investigated in this paper. The sensor was modified with PEDOT:PSS-PB, and two different lactate oxidases (LODs). A rotating disk electrode and Koutecky–Levich analysis were applied for the kinetics analysis and gel optimization. The optimized gel formulation was then tested on disposable screen-printed sensors. The disposable sensors exhibited good performance and presented a high stability for both LOD modifications. Finally, human EBC analysis was conducted from a healthy subject at rest and after 30 min of intense aerobic cycling exercise. The sensor coulometric measurements showed good agreement with fluorometric and triple quadrupole liquid chromatography mass spectrometry reference methods. The EBC lactate concentration increased from 22.5 μM (at rest) to 28.0 μM (after 30 min of cycling) and dropped back to 5.3 μM after 60 min of rest.

## Introduction

In recent years, there has been an increased interest in the lactate concentration for disease diagnostics,^1^ exercise monitoring,^2,3^ and the food industry.^4^ Lactate is produced from pyruvate under anaerobic conditions, causing a lactate acidification of the tissue and blood,^5−7^ potentially leading to decreased tissue oxygenation, left ventricular failure, and drug toxicity.^6^ Higher lactate concentrations are associated with sepsis and septic shock,^1,8,9^ trauma,^10^ tissue hypoxia due to acute lung injury,^11,12^ and respiratory diseases.^7,13^ Lactate levels in the arterial blood range from 0.5 to 2 mM at rest^14^ and can increase to 4 mM for patients with torso trauma^15^ and 2–4 mM for patients with sepsis.^16−18^ Additionally, lactate levels can also act as an indicator in exercise performance. Long-term intense aerobic exercise leads to a rapid increase in lactate levels and results in muscle weakening and fatigue. Lactate levels have been widely used in monitoring athletes’ training and fitness.^19^ One study reported blood lactate levels of 15–25 mM after exercise.^20^

Blood samples, most usually venous blood, arterial blood, or capillary blood sampling, are the traditional diagnostic sample media. However, blood samples are invasive and uncomfortable and are not typically suitable for self-administration or use in non-specialist settings. Performance of the capillary blood monitoring is not suitable for routine clinical use.^21^ The exhaled breath condensate (EBC) to be used in lung and airway diagnostics is a simple and non-invasive method with no potential for adverse effects. Users are typically required to breathe for a few minutes via a tube connected to a collection device, and the exhaled gas, which contains vapor water, mediators, and various ions, is cooled down to a liquid.^22,23^ Lactate in the EBC is not widely used, though one report provides that an EBC lactate level is 21.4 ± 7.7 μM at rest, which is increased to 40.3 ± 23.0 μM after exercise.^5^

Most commercial EBC collection devices can only achieve sample collection, while the analysis is performed by laboratory techniques such as photometry or fluorometry,^24,25^ and mass spectrometry validation is also required.^26^ Refrigeration of EBC samples before laboratory analysis poses the additional risks of sample degradation and additional costs, delays, and logistical barriers to widespread adoption. Disposable sensors are low-cost devices that enable real-time monitoring and rapid measurements.^27^ Therefore, a point-of-care biosensor that is insertable to the condensation device and can analyze the EBC directly after collection would provide a faster, near real-time detection with improved accuracy.

Lactate oxidase (LOD, EC#1.13.12.4) is one of the most common enzymes for amperometric lactate biosensors. Although widely used, the commercially available LOD has an enzyme content of only 10–23%. Therefore, a second, in-house produced LOD was applied for comparison (LOD-N118) that did not contain any additives.

LOD catalyzes the oxidation of lactate to pyruvate in the presence of ambient oxygen and produces hydrogen peroxide (H_2_O_2_),^11,30^ which is directly proportional to the lactate concentration that can be measured by amperometry, via the reaction with Prussian blue (PB).^31,32^ At approximately 0 V versus Ag/AgCl reference electrode, the reduced form of insoluble PB, Prussian White (PW), catalyzes the 2-electron reduction of H_2_O_2_ to water. PB is converted back to PW via electron transfer from the electrode.^33^ PB sensors are easily fabricated and have a high catalytic rate for the H_2_O_2_ reaction and a low enough applied potential (0 V) to avoid most interferents.^34,35^ Maier et al. developed a wearable sensor with PB modification for real-time EBC H_2_O_2_ measurements.^36^ Blending PB with a conducting polymer (CP) has been widely used to increase sensor stability and sensitivity. Curulli et al. demonstrated a poly(1,2-diaminobenzene) nanostructured PB blend,^37^ and Lu et al. developed a polypyrrole–PB blend with higher electroactivity.^38^ In addition, Karyakina et al. illustrated a biosensor modified with LOD (in γ-aminopropyl-triethoxyloxane) and PB.^39^ We report a successful protocol for poly(3,4-ethylenedioxythiophene) poly(styrenesulfonate) potassium ferric ferrocyanide (PEDOT:PSS-PB) based on Chen et al.,^28,40^ where PEDOT:PSS dispersion in the film has been shown to increase the uniformity, charge capacity, and chemical stability of PB nanoparticles.^41^

**Figure 1:**
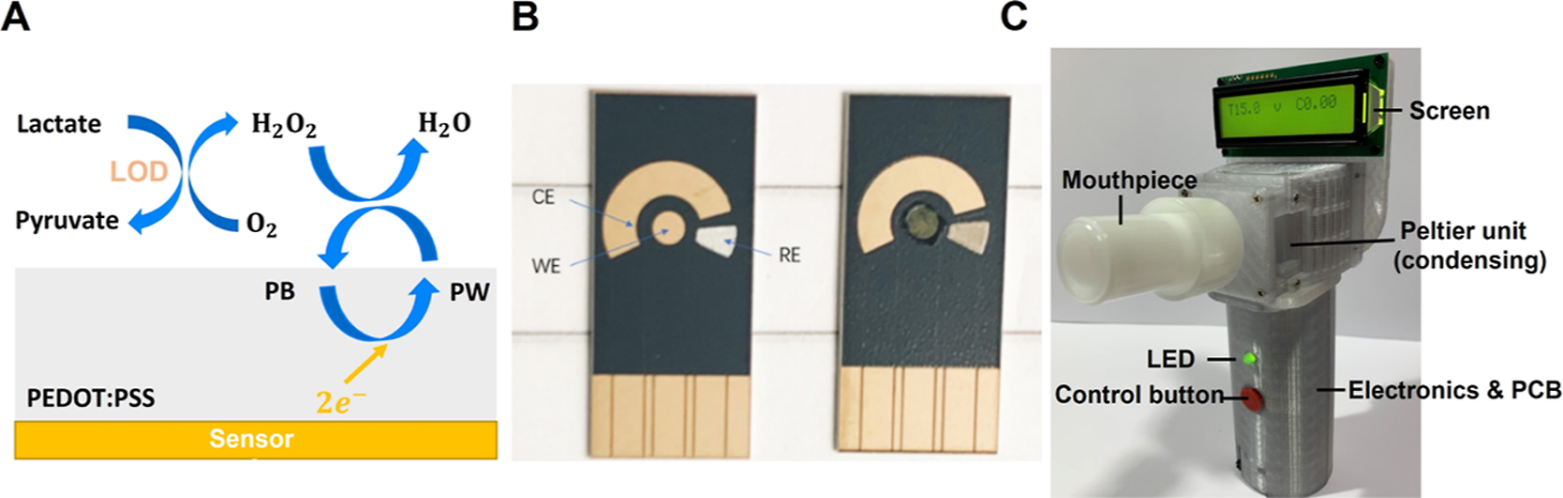
(A) Reaction scheme of the lactate sensor. (B) Blank disposable sensor (L), with PEODT:PSS-PB and LOD modifications (R). (C) EBC collection device (from Respire Diagnostics).^28,29^

In this study, a mixed gel of PEDOT: PSS-PB and LOD (Sekisui Ltd^42^ or in-house N118) was modified on the amperometric biosensor (Figure 1A). A rotating disk electrode (RDE) was employed first for the kinetics study to determine the limiting factor of the reaction. The effect of the enzyme loading and gel membrane thickness on the short-term and long-term stability and sensitivity of the sensor was analyaed, and the optimized gel formulation was transferred to the disposable sensors. Finally, clinical trials were conducted to measure EBC lactate levels from healthy volunteers after intense aerobic exercise, and the results were compared with the results of fluorometric and triple quadrupole liquid chromatography mass spectrometry (TQ LC/MS) reference methods.

## Experimental Section

### Materials

Poly(3,4-ethylenedioxythiophene) polystyrene sulfonate (PEDOT:PSS) (1.3% dispersed in water), lactic acid, 0.1% formic acid, methanol, dimethyl sulfoxide (DMSO), potassium phosphate (monobasic and dibasic), potassium chloride (KCl), bovine serum albumin (BSA), and horseradish peroxidase (HRP) were obtained from Sigma-Aldrich; hydrogen peroxide (30% w/v solution), ferric chloride anhydrous, and potassium ferrocyanide trihydrate were supplied by Fisher Chemical. 10-Acetyl-3,7-dihydroxyphenoxazine (Amplex Red) was supplied by BOC Sciences. The concentrations of all stock standard solutions of H_2_O_2_ were standardized daily using potassium permanganate titration.^44^ Aqueous solutions were prepared from deionized water (DIW), resistance > 18 MΩ cm. Fluorometry 96-MicroWell plates used are MaxiSorp, F96 supplied by Thermo Fisher.

Commercial LOD was obtained from Sekisui Ltd, and LOD-N118 was expressed and purified in-house using *Escherichia coli* shaking flask cultures and immobilized metal affinity chromatography before being freeze-dried in the absence of buffer ions, salts, and stabilizers.

The glassy carbon RDE (radius of 2.5 mm) was obtained from Pine Research (Durham, NC). The disposable sensors were manufactured by Respire Diagnostics (Figure 1B).^29^ The sensor substrate is ceramic-based and consists of a 1 mm diameter gold disk working electrode, an Ag/AgCl reference electrode, and a gold counter electrode. The three parts were separated by laser cutting and insulated with the polymer dielectric grey (Sigma Aldrich) by screen-printing.

### Instrumentation

A potentiostat (CompactStat.h, Ivium technologies, the Netherlands) was used for electrochemical sensor analysis, and a Pine instrument rotator was used for RDE experiments. A fluorometer (Varioskan LUX Multimode Microplate Reader, Thermo Fisher Scientific, UK) was used to validate the sensor measurements using the Amplex Red H_2_O_2_ fluorometry assay. TQ LC/MS (a 1290 Infinity II liquid chromatograph equipped with a pump coupled to an Agilent Ultivo TQ LC/MS system, Santa Clara, USA) was used as the second validation for sensor results. An optical profilometer (Filmetrics Profilm3D, San Francisco, USA) was used to measure the gel membrane thickness on the sensor surface.

### Condensation and Collection of Exhaled Breath

The portable handheld EBC collection device is manufactured by Respire Diagnostics^29^ (Figure 1C). Users exhale through the disposable mouthpiece and the exhaled breath is cooled using a Peltier unit to 20 °C. The trap between the mouthpiece and device minimizes saliva contamination. A volume of 100–150 μL of the EBC is typically collected after 5 min of breathing. The flow rate, humidity, inlet and outlet breath temperatures, and EBC volume for each sample collection are recorded to enable correction for the effects of these factors on the condensation process. At the current stage, EBC samples are pipetted into an Eppendorf tube and stored in the freezer at −20 °C before analysis. A separate electrochemical sensor is used for EBC lactate concentration measurements.

### PEDOT:PSS-PB and LOD Synthesis

This paper follows the Chen and O’Hare PB protocol.^28^ The final composition was as follows: PB (∼43 mM) mixed with PEDOT (0.5% wt %) and PSS (∼43 mM 0.8 wt %). The Sekisui LOD was supplied as a lyophilized powder (30 mg mL^–1^) that was dissolved in potassium phosphate buffer (pH 7.4, 0.1 M). LOD-N118 (60 mg mL^–1^) was supplied in the same potassium phosphate buffer. 94 μL of each LOD solution was crosslinked by 3 mg of BSA, 2 μL of glycerol, and 4 μL of PEGDE to enhance gel stability.^45^ The immobilized LOD gel was then mixed with PEGDE:PSS-PB with a proportion of 1.5:1 by volume, drop-cast onto the working electrode of the sensor, and baked at 55 °C for 2 h. The effect of the LOD concentration on the sensor activity, stability, and sensitivity was investigated.

### RDE Preparation

A glassy carbon RDE from Pine Research was used to identify the limiting factor of the electrode reaction and investigate the gel effect on the sensor performance. RDE is a steady-state technique and is therefore not affected by current contributions from double layer charging. Furthermore, independent control of potential and mass transport enables separation of kinetics from mass transport effects and the elucidation of the mechanism using the well-established Koutecky–Levich analysis.^46,47^

Different concentrations of lactate (50–250 μM) were added to the testing electrolyte (0.1 M potassium phosphate buffer + 0.1 M KCl), and for each concentration, the steady-state current was recorded for the RDE rotating at speeds ranging from 49 to 400 rpm. Similar experiments were conducted on H_2_O_2_ as a comparison, which indicated the conversion rate from lactate to H_2_O_2_ and therefore the estimation of the real lactate concentration.

### Koutecky–Levich Analysis

There are several possible resistances that can limit the rate of the sensor reaction: (i) the transport of lactate in the external diffusion boundary layer above the coated electrode surface , (ii) the transport of lactate in the film membrane , (iii) transport of H_2_O_2_in the film membrane , (iv) the kinetics of the lactate–LOD enzyme reaction , (v) the PB–H_2_O_2_ catalytic reaction , and (vi) the electrode reaction for transformation of PW to PB . Thus, the overall current can be represented as the reciprocal sum of these resistances by the Koutecky–Levich equation
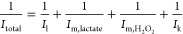
1*I*_l_ is the current when limited totally by mass transport in the liquid boundary layer described by the Levich equation, and eq 1 then becomes
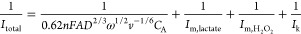
2

The Koutecky–Levich equation is analyzed by plotting the reciprocal current against the reciprocal square root of the RDE rotation rate in radians per second (). Therefore, the slope of the Koutecky–Levich plot represents the mass transport in solution (*i*_l_), while the intercept information represents any combination of the remaining factors.

When a diffusion limited potential is applied for amperometry,  = 0. As the PB catalytic reaction and PB–PW transformation are rapid at all potentials needed, the terms  and  are negligible as well. Hence, the Koutecky–Levich intercept components are reduced to (i) mass transport of lactate in the film membrane , (ii) mass transport of H_2_O_2_in the film membrane , and (iii) kinetics of the enzyme reaction , whose equations are represented in Table 1 (for the meaning of symbols, see Supporting Information Table S1).

**Table 1:** Summarized RDE Limiting Factors

limiting factor	description	equation
(K–L slope)	the transport of lactate in the external diffusion boundary layer above the coated electrode surface	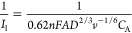
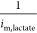	mass transport of lactate in the film membrane	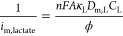
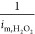	mass transport of H_2_O_2_ in the film membrane	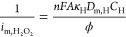
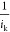	kinetics of the LOD enzyme reaction	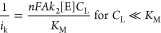
overall intercept current	=	

### Disposable Sensor for Real Sample Analysis

Disposable sensors modified with the same procedure as that for the RDE were applied in the real EBC lactate analysis. The samples were obtained from healthy volunteers at rest and after exhausting aerobic exercise. 50 μL of potassium phosphate buffer (pH 7.4, 0.1 M) with KCl (0.1 M) was used as a testing electrolyte. A series of lactate solutions (6.25, 12.5, 25, 50, and 100 μM) were then dropped onto the sensor surface for a calibration. 50 μL of the EBC samples was mixed with a salt made from the same buffer due to the EBC composition and dropped onto the sensor.

For each sample, amperometry was applied at −0.15 V for 180 s. The chronoamperometric response to a mass transport limited potential step was applied, and the induced current was proportional to the H_2_O_2_/lactate concentration,^48^ following the Cottrell equation (eq 3). Coulometry integrates the chronoamperometric current over time, which smooths the random noise from the original signal.^49^ The edge effect on the circular planar disk electrode is also described by digital simulation by Flanagan and Marcoux (eq 4).^50^
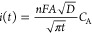
3
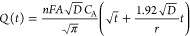
4

### Clinical Experiment Protocol (IRAS ID 274161)

Ethical approval for the healthy volunteer study (*n* = 7) was granted by the London–Bloomsbury Research Ethics Committee (reference 20/LO/0364). Healthy volunteers undertook intense cycling on an exercise bicycle for 30 min, starting from 35 W, with an increase in the power of 35 W every 5 min up to the maximum of 210 W. Before the exercise, one EBC sample was collected, and the participant was required to rinse the mouth before collection to minimize saliva contamination and then to exhale for 5 min through the EBC device. Another EBC sample was collected immediately after the exercise, and four more samples were taken in the next 60 min rest. All experiments were conducted at a room temperature of 25 °C. A more extensive analysis will be the subject of a subsequent publication, but typical exemplary data are discussed below.

### Amplex Red Fluorometry Hydrogen Peroxide Assay

Amplex Red reacts with H_2_O_2_ in the presence of HRP and produces resorufin, which can be detected fluorometrically with maximal excitation and emission wavelengths at approximately 571 and 585 nm, respectively.^51^ The Amplex red assay was used as a comparison and validation for sensor measurements (eq 5). Amplex Red (2.5 mM) was dissolved in DMSO and stored at −20 °C before use. HRP (5 U mL^–1^) was mixed with PBS (pH 7.4, 0.1 M) and stored at −20 °C before use. LOD solution was further diluted to 5 U mL^–1^ by the addition of potassium phosphate buffer (pH 7.4, 0.1 M). For assaying, 46 μL of the analyte was prepared into a 96-well plate with the addition of 2 μL of Amplex Red stock solution, 1 μL of HRP stock solution, and 1 μL of LOD (5 U mL^–1^). The excitation and absorption wavelengths were set to 535 and 587 nm, respectively.
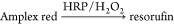
5

### LC/MS Assay

The TQ LC/MS method was used as a second reference method. Formic acid (0.1%) in methanol (10%) (mobile phase) and a reverse-phase analytical column (50 mm by 4.6 mm; Kinetex 5 μm FA) were used. The eluent flow rate was 0.4 mL min^–1^, and the injection volume was 3 μL. The electrospray interface (ESI) was in use for the mass spectrometric detection in the negative multiple reaction monitoring (MRM) mode for the detection of lactate. Triple quadrupole detector transitions were used for quantification and qualification as follows: ion mass/charge ratio (*m*/*z*) = 89 M^–1^ and *i*_*m*/*z*_ = 71:43:10. Optimized parameters for ESI and MS were found to be with a capillary voltage of 4.5 kV, a nozzle voltage of 1.5 kV, a gas temperature of 200 °C, a gas flow of 11 L min^–1^, and a sheath gas flow of 12 L min^–1^.

## Results and Discussion

### RDE and Koutecky–Levich Analysis

**Figure 2:**
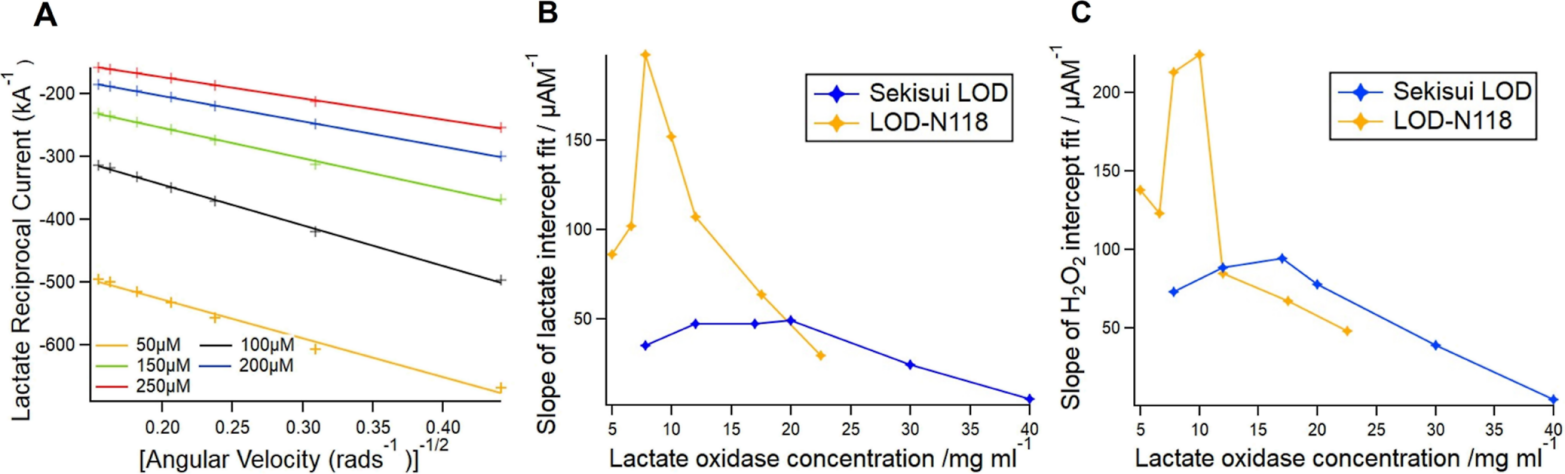
(A) Koutecky–Levich plot for the RDE modified with PEDOT:PSS-PB and Sekisui LOD for lactate concentrations between 50 and 250 μM. (B) Slope of the K–L intercept fit against LOD concentration for lactate measurements and (C) slope of the K–L intercept fit against LOD concentration for H_2_O_2_ measurements.

From cyclic voltammograms (Supporting Information Figure S1), a mass transport-controlled current is observed for a potential of <−0.15 V, which is therefore applied in amperometry. Figure 2A demonstrates a linear Koutecky–Levich plot for several lactate concentrations smaller than *K*_M_ (0.5 mM). The detection limits for the RDE modified with Sekisui LOD and LOD-N118 are 78.3 and 465.0 nM, respectively.

### Diffusivity in Solution

From Koutecky–Levich slopes (Supporting Information Figure S2), diffusion coefficients of lactate (*D*_l_) and H_2_O_2_ (*D*_H_) in bulk solution are calculated (Table 2). These data are consistent with the published results.^28^

**Table 2:** Diffusion Coefficients of Lactate and H_2_O_2_in Bulk Solution for the RDE Modified with Sekisui and LOD-N118, Compared with Published Results

	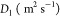	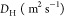
Sekisui	(7.31 ± 0.69) × 10^–10^	(1.16 ± 0.05) × 10^–9^
LOD-N118	(7.10 ± 0.12) × 10^–10^	(1.40 ± 0.17) × 10^–9^
Oyaas et al.^52^	6.90 × 10^–10^	
Chen and O’Hare^28^		1.49 × 10^–9^

### Mass Transport Rate Constant in the Gel Membrane

Since the partition coefficient (κ) is unknown, the mass transport rate constant (*k*_m_) in the film is estimated instead of diffusion coefficients of lactate and H_2_O_2_ in the film (from the K–L intercept) and is dependent on the current that is limited totally by mass transport in the film membrane (*I*_f_) and film thickness (ϕ) (eq 6). Table 3 shows the effect of film thickness (optical profilometer measurements) on *k*_m_ values. Thicker films were found for higher LOD concentrations. Constant *k*_m_ values were estimated for film thickness between 7.3 and 20.5 nm, while *k*_m_ decreases for films of higher thickness (20.5–43.3 nm).
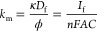
6

**Table 3:** Film Thickness and Lactate Diffusion Coefficient in the Film for Different Sekisui LOD Concentrations on the RDE

[LOD] (mg mL^–1^)	film thickness (nm) (*n* = 3)	mass transport rate constant (*k*_m_) (ms^–1^)
8	3.2	9.18 × 10^–6^
12	7.3	1.24 × 10^–5^
16	10.2	1.24 × 10^–5^
20	20.5	1.29 × 10^–5^
30	28.9	6.32 × 10^–6^
40	43.3	1.29 × 10^–6^

### Optimization of the LOD Gel

The effect of Sekisui LOD and LOD-N118 concentrations in the gel matrix on the RDE performance is analyzed from the intercept data of K–L plots (Figure 2B,C). For the Sekisui enzyme, at a low LOD concentration (8 mg mL^–1^), the enzyme kinetics () becomes the rate-limiting step. For the LOD concentration in the range between 12 and 20 mg mL^–1^, the intercept current is independent of LOD concentration and is consistent with the excess enzyme, and the lactate reacts with LOD instantly at the enzyme gel surface. Therefore, the  term and  term are negligible within this range, and the rate-determining step of the reaction is H_2_O_2_ diffusion in the gel membrane (. However, as the LOD concentration increases (30 mg mL^–1^), the intercept current decreases. H_2_O_2_ calibrations show similar trends to those for lactate for both enzymes. There are two possible interpretations for the decreasing intercepts: (i) significantly high LOD concentrations led to an increase in film thickness and therefore affected the H_2_O_2_ mass transport rate constant *k*_m_ (Table 3) and (ii) H_2_O_2_ is decomposed chemically by the enzyme, possibly via the flavin mononucleotide (FMN, the quinoid prosthetic group in LOD), or the protein itself, depending on the enzyme concentration, rate constants, and thermodynamics (Supporting Information Table S2). LOD-N118 demonstrates a similar performance the intercept current increases in the 5 to 8 mg mL^–1^ concentration range and then decreases for higher LOD concentrations. It is noteworthy that the LOD-N118 is a purer product, and therefore, this phenomenon ought to manifest at lower concentrations (Figure 2B,C).

We want the reaction to be mass transport-limited to minimize the effect of enzyme kinetics. Therefore, the point for the largest intercept is chosen as the optimal LOD concentration to control the reaction to be limited by the mass transport (20 mg mL^–1^ for Sekisui LOD, 7.8 mg mL^–1^ for LOD-N118). The same gel composition is selected for the disposable sensor against surface area.

### Comparison between Sekisui and LOD-N118

As the reaction is limited by the enzyme kinetics at low LOD concentrations, the catalytic rate constant (*k*_2_) of LOD is estimated from the Koutecky–Levich intercept at the lowest LOD concentrations (5 mg mL^–1^ for LOD-N118 and 7.8 mg mL^–1^ for Sekisui LOD), where the intercept is dominated by the kinetics-limited current (), and with known *K*_M_ values (0.5 mM for LOD-N118 and 0.7 mM for Sekisui LOD). LOD-N118 demonstrated an ∼ 4.3 times higher *k*_2_ value (1.05 × 10^–5^ s^–1^) than Sekisui LOD (2.45 × 10^–6^ s^–1^), which represents a higher activity, despite both enzymes having comparable specific activities (18 U mg^–1^ for LOD-N118 and >20 U mg^–1^ for Sekisui LOD). Furthermore, LOD-N118 was used at half the concentration of Sekisui LOD. The higher activity by LOD-N118 is attributed to its lack of additives and therefore the higher content of the active enzyme (Sekisui LOD contains 77–90% additives).

### Disposable Electrochemical Sensor

**Figure 3:**
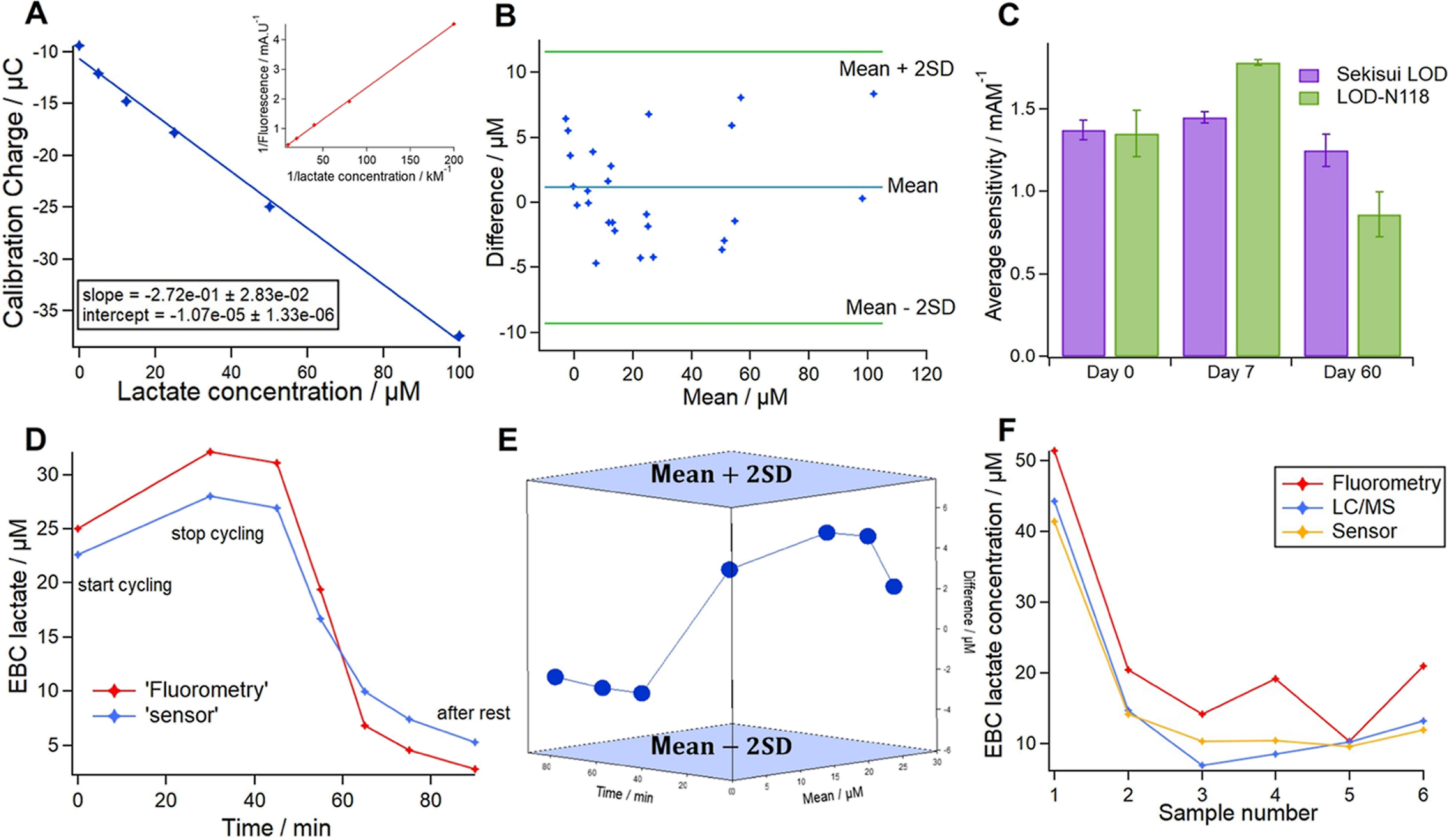
(A) Coulometry calibration measured using the electrochemical sensor with PEDOT:PSS-PB-LOD modification, against the Lineweaver–Burk plot for fluorimetry calibration using the Amplex red and HRP assays. (B) 2D Bland Altman analysis comparing electrochemical sensor results and the Amplex Red assay results (*n* = 5). (C) Comparison of the sensor average sensitivity (± sd, *n* = 3) with Sekisui LOD and LOD-N118 gel modifications at day 0, 7, and 60. (D) Sensor and fluorometric data for EBC lactate concentrations measured from a healthy subject at rest, directly after exercise, and after 45 min of rest. (E) 3D Bland–Altman analysis comparing sensor and fluorometric results. (F) EBC lactate measurements from a healthy volunteer at rest using a sensor, TQ LC/MS, and fluorometry.

Figure 3A shows a linear plot for sensor calibration with Sekisui LOD modification for lactate concentrations ranging from 0 to 100 μM. As a reference method, a fluorometric calibration with 0–100 μM lactate measured in solution is illustrated by a Lineweaver–Burk plot, which draws a linear calibration between the reciprocal fluorescence signals and reciprocal lactate concentrations. Bland–Altman analysis (Figure 3B, *n* = 5) shows good agreements between the sensor and fluorometry calibrations.

### Short-Term and Long-Term Stability

Immobilization of the LOD gel with glycerol, PEGDE, and BSA enhanced the stability of the sensor since the gel without crosslinking is easily peeled off from the sensor surface after a few days. The lifetime for the immobilized sensor is 2 days at room temperature, 1 week at +4 °C storage, and over 6 months at −20 °C storage.

Sekisui LOD requires a shorter stabilizing time than LOD-N118 before use. Sekisui LOD usually takes one–two transients (one transient represents a 180 s amperometry measurement for one lactate concentration), while LOD-N118 requires five–six transients and has a larger current response than Sekisui LOD. Sensors with LOD-N118 modification have a higher detection limit of 29.3 nM than those with Sekisui LOD (5.0 nM) due to the higher background signal.

Figure 3C shows the sensitivity comparison for Sekisui LOD and LOD-N118 modified sensors (*n* = 3) tested at day 0, day 7, and day 60. At day 0, both enzymes demonstrate a consistent sensitivity (1.36 mA M^–1^) and a high current response, which can achieve 80% of the theoretical value (from the Cottrell equation). The sensitivity of Sekisui LOD remains constant over 7 days and slightly decreases to 85.1% after 60 days (*n* = 3) compared with day 0. In contrast, LOD-N118 exhibits a noticeable increased sensitivity of up to 130% after 7 days but a decreased sensitivity of 56.9% after 60 days (*n* = 3). These higher variations of sensitivity from LOD-N118 upon storage might again be explained by the absence of additives that interact with the the sensor architecture and are not yet optimized. We can conclude that both Sekisui LOD and LOD-N118 exhibit high stability and sensitivity in the short term, while LOD-N118 shows lower stability in long-term storage.

### EBC Lactate Sample Analysis

Figure 3D demonstrates the EBC lactate levels measured using sensor and fluorometric calibrations from a healthy subject at rest and after 30 min of cycling (power ranges between 35 and 210W). The 3D Bland–Altman plot in Figure 3E shows a reasonable agreement between sensor and fluorometry measurements over the physiologically relevant range and is independent of time since all differences fall within the 95% confidence limits within the time series.

The EBC lactate level increased from 22.5 μM (at rest) to 28.0 μM (after the maximal exercise load) and dropped back to 5.3 μM after 60 min of rest. Data from other volunteers show a similar trend that the EBC lactate level increased during exercise and then decreased afterward. The initial EBC lactate levels before exercise are always higher than those at rest afterward. To validate sensor measurements, LC/MS is used as a second reference method. A good correlation between sensor, fluorometry, and LC/MS measurements was found (Figure 3F). Therefore, the decreasing trend does not originate from the analytical methods and is more likely to be buccal in origin. One publication shows an increasing saliva lactate level after eating, which may influence the initial EBC lactate levels.^53^ Lactate production in dental biofilms may be another contamination source for EBC lactate.^54^ Future research will focus on clinical protocol optimization to minimize contamination.

## Conclusions

In this study, we developed a PEDOT:PSS-PB-LOD gel on gold disposable sensors, able to measure lactate in the EBC under physiological conditions. The enzyme gel formulation was optimized based on RDE experiments and kinetics analysis, investigating the effect of enzyme concentration and gel thickness, and was applied to disposable sensors, which exhibited high stability and sensitivity. Using LOD-N118 without additives resulted in higher currents but also a higher variation upon storage, compared to those of commercial Sekisui LOD. The enzyme gel formulation was optimized based on RDE experiments and kinetics analysis, investigating the effect of enzyme concentration and gel thickness, and was applied to disposable sensors, which exhibited high stability and sensitivity in both short-term and long-term tests. Human EBC analysis was conducted for a healthy subject at rest and after 30 min of intense aerobic cycling exercise. Coulometry results of the sensor showed a good correlation with the results of fluorometry and TQ LC/MS reference methods. Measured EBC lactate concentrations increased from 22.5 μM (at rest) to 28.0 μM (after 30 min of cycling) and dropped back to 5.3 μM (after 60 min of rest).

## Ethical Approval

Ethical approval for the healthy volunteer study was granted by the London–Bloomsbury Research Ethics Committee (20/LO/0364) Participants gave informed consent to participate in the study before taking part. The study was sponsored by Imperial College London and conducted at the National Institute of Health Research/Wellcome Trust Imperial Clinical Research Facility (Imperial College London, UK). All researchers underwent Good Clinical Practice training, and procedures were conducted in accordance with the 1964 Declaration of Helsinki and later amendments.

## Supporting Information Available

Meaning of symbols in equations; cyclic voltammograms for RDE analysis; Koutecky–Levich plots for PEDOT:PSS-PB-LOD gel modification on the RDE; effect of H_2_O_2_ and LOD concentrations on gel optimization by fluorometry; and calculation of fabrication costs for the EBC device and sensor (PDF)
