# Supplementary material for: Rapid Measurement of Lactate in the Exhaled Breath Condensate: Biosensor Optimization and In-Human Proof of Concept
Source: ACS Sens. 2022 Nov 21;7(12):3809–16. doi: 10.1021/acssensors.2c01739 (PMC9791687; doi:10.1021/acssensors.2c01739)
Supplement: Supplementary file 1 — se2c01739_si_001.pdf [file se2c01739_si_001.pdf]

Supporting Information

Rapid measurement of lactate in exhaled breath condensate: biosensor optimisation and in-human proof-of-concept

Shulin Zhang*, Yu-chih Chen*, Alaa Riezk†, Damien Ming†, Lidiia Tsvik~, Leander Sützl~, Alison Holmes†, Danny O'Hare*

* Department of Bioengineering, Imperial College London, London SW7 2AZ, United Kingdom

† Faculty of Medicine, Department of Infectious Disease, Centre for Antimicrobial Optimisation, Imperial College London, London SW7 2AZ, United Kingdom

~ Laboratory of Food Biotechnology, Department of Food Science and Technology, BOKU-University of Natural Resources and Life Sciences Vienna, Muthgasse 11, A-1190 Wien, Austria

Table of contents

Meaning of symbols in equations. S-2
Cyclic voltammograms for RDE analysis. S-3
Koutecky-Levich plots for PEDOT: PSS-PB-LOD gel modification on RDE. S-4
Effect of H_2O_2 and LOD concentrations on gel optimisation by fluorimetry S-5
Calculation for the fabrication costs of the sensor and the EBC device. S-6

1. Meaning of symbols in equations

Symbol	Meaning	Units
n	The number of moles of electrons transferred in the half reaction	$number$
F	Faraday's constant	$C\ mol^{-1}$
A	RDE surface area	m^2
D	Analyte diffusion coefficient in solution	m^2s^{-1}
D_L	Lactate diffusion coefficient in solution	m^2s^{-1}
D_H	H_2O_2 diffusion coefficient in solution	m^2s^{-1}
D_m	Analyte diffusion coefficient in film membrane	m^2s^{-1}
$D_{m,L}$	Lactate diffusion coefficient in film membrane	m^2s^{-1}
$D_{m,H}$	H_2O_2 diffusion coefficient in film membrane	m^2s^{-1}
ν	Kinematic viscosity of analyte in solution	m^2s^{-1}
C_A	Analyte concentration	$mol\ l^{-1}$
C_L	Lactate concentration	$mol\ l^{-1}$
C_H	H_2O_2 concentration	$mol\ l^{-1}$
κ_L	Partition coefficient of lactate	-
κ_H	Partition coefficient of H_2O_2	-
ϕ	Membrane film thickness	m
k_2	Catalytic rate of the rate limiting step	s^{-1}
K_M	Michaelis-Menten constant	$mol\ l^{-1}$
$[E]$	LOD enzyme concentration	$mol\ l^{-1}$

Table S1. Meaning of symbols in equations

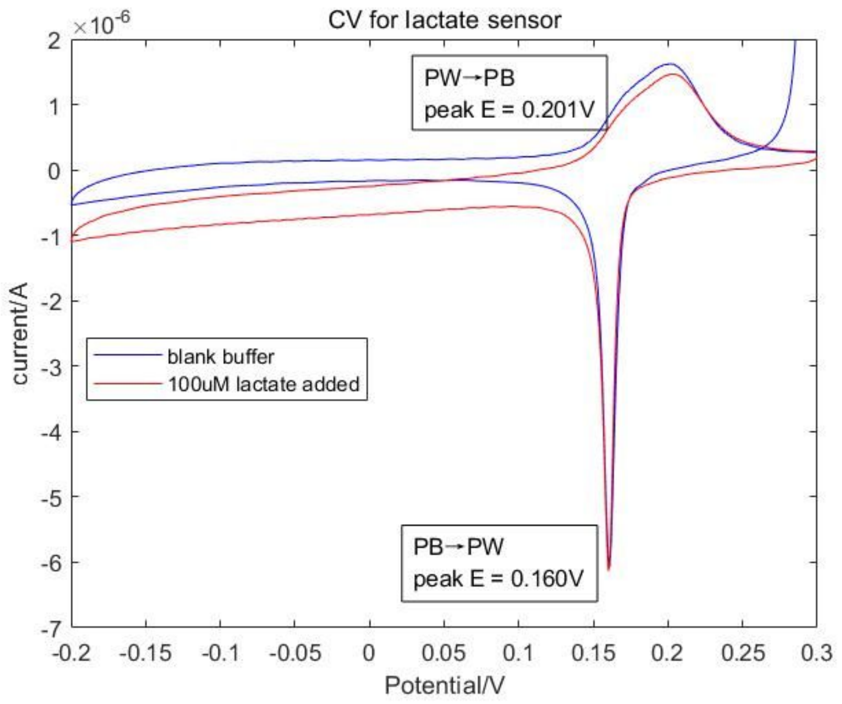

(A)

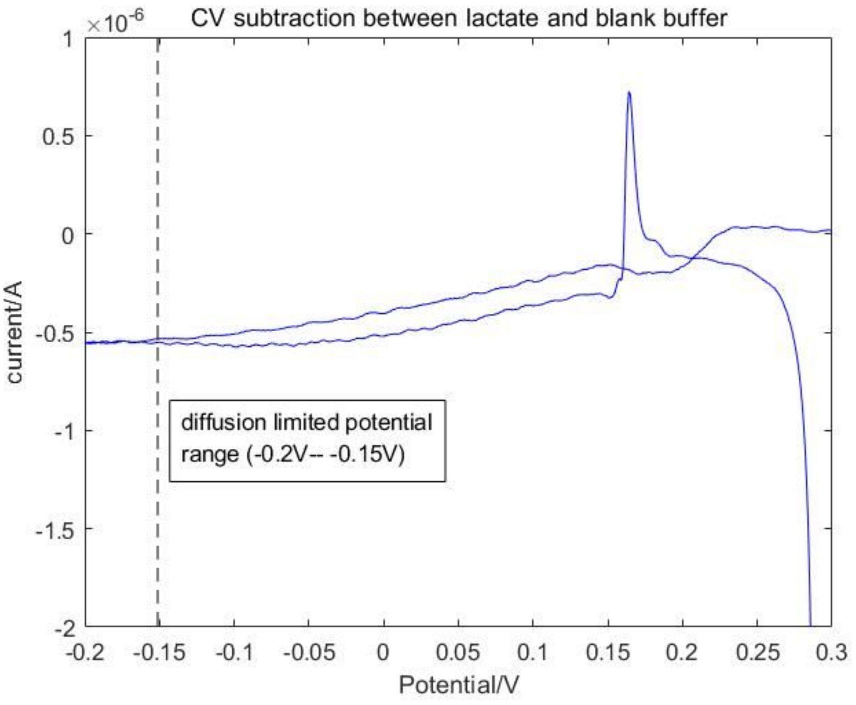

(B)

Fig. S1 Cyclic voltammograms for RDE analysis. (A) Cyclic voltammogram for RDE modified with PEDOT:PSS-PB and Sekisui LOD in blank buffer and in 100 μM lactate. Reduction and oxidation peaks are at 0.160V and 0.201V, respectively. (B) Subtraction of the two CVs, giving the mass transport controlled current between -0.2V and -0.15V, where -0.15V is applied in amperometry measurements.

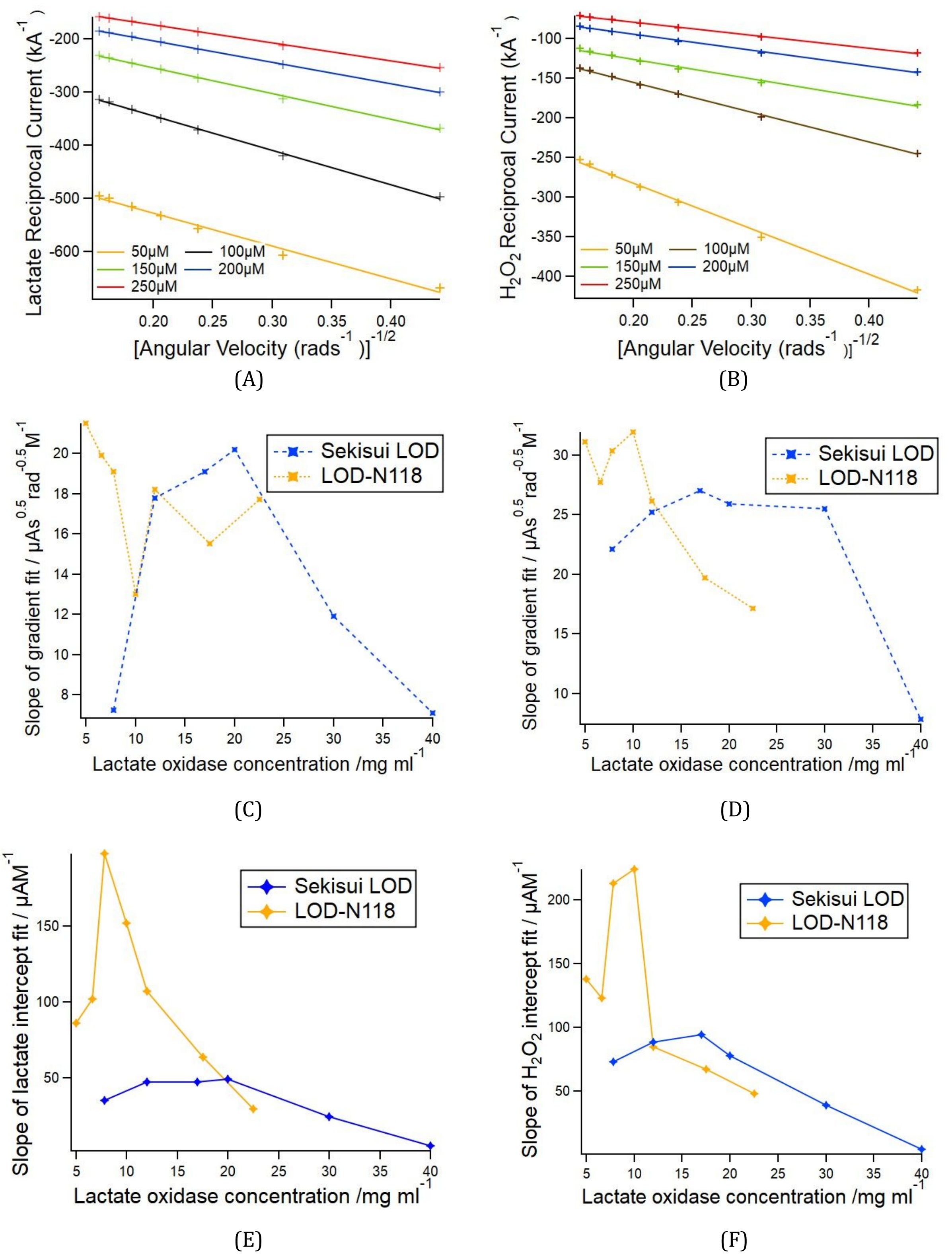

Figure S2. Koutecky-Levich (KL) plots on RDE modified with Prussian blue and LOD (Sekisui) (A)for lactate, (B) for H_2O_2 measurements. (C) Reciprocal slope of KL plot of different LOD concentrations against lactate concentrations, (D) against H_2O_2 concentrations.

(E) Reciprocal intercept of KL plot of different LOD concentrations against lactate concentrations, (F) against H_2O_2 concentrations.

$$(Enzyme)FMNH_2 + H_2O_2 \rightarrow FMN + H_2O \quad (8)$$

$$(Enzyme)FMN + H_2O_2 \rightarrow FMNH_2 + O_2 \quad (9)$$

[LOD] μM / [H_2O_2] / μM	50	100	200	300	400	500
0	41.9	43.9	49.4	41.8	35.6	61.7
31.25	1105	976.5	1029	940.6	828.6	809
62.5	1685	1655	1393	1415	1278	1178
125	2116	2006	1862	1848	1751	1625
250	2026	1709	1628	1573	1515	1507
500	1729	1834	1577	1451	1438	1450

Table S2. Effect of H_2O_2 and LOD concentrations on gel optimisation by fluorimetry. This table shows the fluorometric signal with Amplex Red and HRP assay for different H_2O_2 and LOD concentrations. The results demonstrate a decreasing fluorometric signal for both high LOD and H_2O_2 concentrations, which prove that H_2O_2 is decomposed chemically by the enzyme, possibly via the flavin mononucleotide (FMN), the quinoid prosthetic group in LOD, for both significantly high LOD and H_2O_2 concentrations (Eqn 8 Eqn 9).

Item	Expense / $
**EBC Device**	
Integrated circuit	150
PCB	100
Components	200
Outer shell	150
**Overall cost per device**	**600**
**Disposable sensor**	
bare sensor	1.5
Lactate oxidase (1 g) (CAT#RELO-70-1381)	765
Lactate oxidase per sensor (0.054 mg)	0.041
**Overall cost per sensor**	**1.541**

Table S3. Fabrication costs of EBC device and disposable sensor. Both EBC device and bare sensor are manufactured and produced by Respire Diagnostics, whilst many other chemicals and reagents are produced in-house and therefore not included. Laboratory costs are not included at this stage since they do not reflect commercial manufacturing costs. Components and commercially purchased reagents are listed with prices or catalogue number where available.
